# Migration of a ureteral stent made from a nasogastric tube into the inferior vena cava: A case report^☆^

**DOI:** 10.1016/j.ijscr.2024.110786

**Published:** 2024-12-26

**Authors:** Solomon Bekele, Biruk T. Mengistie, Chernet T. Mengistie, Solyana Bereded, Asteway M. Haile, Telila K. Belisa

**Affiliations:** a Department of Surgery, Addis Ababa University, Addis Ababa, Ethiopia; b School of Medicine, College of Health Sciences, Addis Ababa University, Addis Ababa, Ethiopia

**Keywords:** Foreign body, Nasogastric tube, Inferior vena cava

## Abstract

**Introduction:**

This case report presents a rare instance of a ureteral stent, fashioned from a nasogastric tube, migrating into the inferior vena cava (IVC). The report underscores the importance of timely diagnosis and intervention to prevent severe complications.

**Presentation of case:**

A 38-year-old woman presented with a ureteral stent, made from a nasogastric tube, found in the IVC following a previous surgery. The foreign body was surgically retrieved, and the patient had an uneventful recovery.

**Discussion:**

Endovascular foreign bodies pose significant risks and should be promptly removed to prevent severe complications. In this case, surgical intervention was chosen due to the lack of available endovascular instruments and expertise.

**Conclusion:**

Timely surgical intervention and vigilant monitoring are crucial for managing intravascular foreign bodies to ensure successful outcomes.

## Highlights

• Rare case of a nasogastric tube migration into the inferior vena cava
• Successful surgical retrieval of the intravascular foreign body without complications
• Demonstrates the critical role of imaging in diagnosing intravascular foreign bodies
• The case underscores the need for prompt intervention to prevent severe complications.

## 1 Introduction

The increasing use of endovascular prostheses and improvised medical devices has led to a rise in cases of foreign body migration into the vasculature. While intravascular migration is a rare complication, it poses significant risks, including thrombosis, embolization, and infection [1,2]. Migration of nasogastric tubes (NGTs) into the vascular system is exceedingly uncommon, with limited cases documented in the literature. Proposed mechanisms include vessel wall erosion, improper placement, or inadvertent entry into vascular structures [3,4].

Intravenous foreign body migration has been reported as early as 1834 [5]. Such complications necessitate swift diagnosis and intervention due to their potential for life-threatening outcomes. This report highlights a rare case of a ureteral stent fashioned from an NGT migrating into the inferior vena cava (IVC). By focusing on the mechanisms of migration, diagnostic challenges, and management strategies, this case provides valuable insights into handling such rare and critical complications. This manuscript was prepared following the SCARE Guidelines 2023 criteria [10].

## 2 Case report

A 38-year-old woman was referred to our hospital two months after undergoing an exploratory laparotomy and right hemicolectomy with ileo-transverse anastomosis for generalized peritonitis due to cecal perforation. During the initial surgery, a right ureteric injury was identified, likely caused by the extensive inflammation and tissue friability associated with cecal perforation and generalized peritonitis. Repair was attempted using a 4Fr nasogastric tube (NGT) as a temporary ureteral stent. This improvisation was necessitated by the unavailability of standard ureteral stents at the time and was intended to maintain ureteral patency and facilitate healing. The stent was secured with the expectation that it would remain in place until cystoscopic removal, which was scheduled postoperatively.

**Fig. 1:**
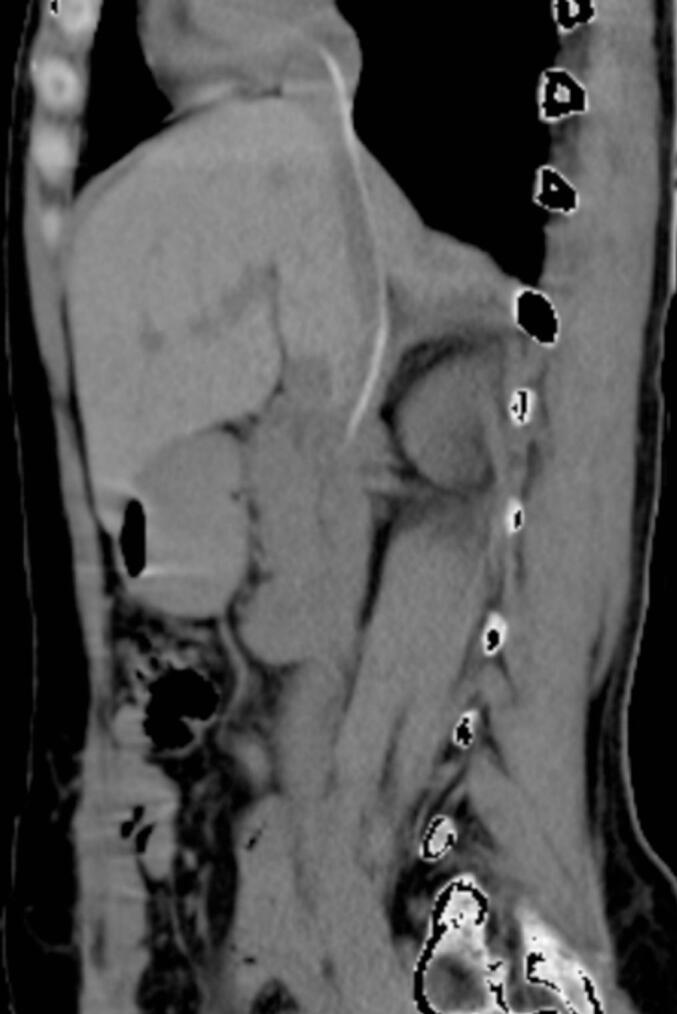
CT scan showing radio-dense tubular foreign body in the IVC.

The patient's immediate postoperative course was uneventful, and she was stable upon discharge. However, during the planned cystoscopic procedure for stent removal, the NGT could not be located. An intravenous urography (IVU) was initially performed, but the results were normal, with no visible foreign body. A subsequent contrast-enhanced CT scan (Fig. 1) revealed a radiopaque tubular foreign body located within the inferior vena cava (IVC). Echocardiography showed normal cardiac function, with echogenic material observed in the right atrium. Laboratory findings, including white cell count, liver function tests, and renal function tests, were within normal ranges.

The patient was taken to the operating room for surgical retrieval of the foreign body. An upper midline incision was performed, followed by adhesiolysis and extended Kocherization to expose the IVC. The foreign body (4Fr NGT) was palpated inside the IVC and carefully removed after a small venotomy was performed. During the retrieval, no thrombus or fistulous connection between the ureter and venous system was identified, ruling out this as a potential migration route. Additionally, the surrounding tissue demonstrated no significant inflammatory or fibrotic changes. The venotomy site was repaired using 5–0 prolene sutures. The patient had an uneventful postoperative course and was discharged on the fifth postoperative day with no complications noted on follow-up.

## 3 Discussion

Foreign body migration into the venous system is a rare but serious complication. The risks associated with retained foreign bodies in the IVC include thrombosis, embolization, and septicemia. Timely retrieval is essential to prevent these outcomes [5,6].

Foreign bodies enter the vascular system by penetrating the wall, then migrate with the help of gravity or blood flow. The usual path for migration of foreign bodies is from the peripheral veins to the right side of the heart, this has been described in various literatures [5]. Asymptomatic patients with a foreign body in their vascular system have also been reported. Rarely, foreign bodies can migrate in a retrograde path from the heart to peripheral vessels [6]. In our patient, the NGT is hypothesized to have migrated into the inferior vena cava via the right gonadal vein, likely due to inadvertent entry during the initial surgery. The patient's cecal perforation and resulting inflammation could have contributed to vessel wall fragility, increasing the likelihood of such an occurrence. The absence of a thrombus or fistulous connection further suggests direct entry into the venous system.

Knowing where exactly the foreign body is located in the vascular system is of utmost importance in planning retrieval. The exact location of a foreign body can be determined through echocardiography and/or CT. Chest radiography gives valuable information when it's taken in different views [7]. In our patient, a CT scan revealed a radiopaque, longitudinal foreign body in the Inferior vena cava, extending from the site of renal veins with its tip coiled in the right atrium.

Vascular foreign bodies should always be removed, even in asymptomatic patients, because it can cause obstruction to major vessels, and have a risk to embolize, erode and cause bleeding. Even though the complications of a retained vascular foreign body reported by various literatures reach 25 %, retrieval with recent techniques is very safe [8]. Retrieval using endovascular techniques is the current standard of practice recommended given the forgiven body is smooth and there is no contamination. Whereas foreign bodies with sharp edges in the vascular system usually cause complications [9]. Endovascular retrieval offers a minimally invasive approach with high success rates. It also offers a shorter recovery time, reduced risk of infection, and avoidance of major surgical trauma [8]. However, in this case, the lack of endovascular instruments and expertise necessitated open surgical retrieval.

In contrast, surgical retrieval provides the advantage of direct visualization and control of the affected vasculature. In this case, it allowed the surgeon to address the presence of adhesions around the IVC and repair the venotomy with prolene sutures, ensuring no residual injury or thrombosis. The primary disadvantage of surgical retrieval is its invasiveness, which may increase recovery time and associated risks, such as bleeding or infection [9].

The location of the foreign body within the IVC poses specific risks, including obstruction of venous return, pulmonary embolism, and potential erosion into adjacent structures [10]. In this patient, the stent's proximity to the right atrium heightened the risk of embolic events and cardiac complications. Comprehensive imaging and meticulous surgical planning are crucial to minimizing these risks. Whenever available, interventional radiology with endovascular procedures and proper device selection should be the management choice. Endovascular retrieval done with experienced personnel avoids procedure-related complications [10].

## 4 Conclusion

Vascular foreign body removal largely depends on its location, available resources, and expertise. Various techniques can be utilized for this approach, but patient-based management should be planned for optimal outcomes. This case underscores the importance of careful device placement and follow-up, particularly when using improvised medical devices. Surgeons should maintain a high index of suspicion for device migration and perform thorough evaluations in patients presenting with unexplained symptoms. Prompt imaging and appropriate intervention, whether endovascular or surgical, are essential for managing intravascular foreign bodies effectively.

## CRediT authorship contribution statement

**Solomon Bekele:** Pre-operative preparation, operation, post op follow-up, conceptualization and writing the original draft, guarantor.
**Biruk T. Mengistie:** Writing the original draft, resources, data curation, guarantor.
**Chernet T. Mengistie:** Writing the original draft, visualization, writing – review & editing, guarantor.
**Solyana Bereded:** Supervision, writing – review & editing.
**Telila K. Belisa:** Writing – review & editing, data curation.
**Asteway M. Haile:** Writing – review & editing, visualization.

## Ethical approval

IRB review and approval were waived for this case report.

## Guarantor

Solomon Bekele
Biruk T. Mengistie
Chernet T. Mengistie.

## Research registration number

N/A.

## Consent for publication

Written consent was obtained from the individual patient.

## Funding

No funding was provided for the completion of this manuscript.

## Declaration of competing interest

The authors declared that there are no conflicts of interest to disclose.
